# Supplementary material for: The experiences of adult heart, lung, and heart-lung transplantation recipients: A systematic review of qualitative research evidence
Source: PLoS One. 2020 Nov 11;15(11):e0241570. doi: 10.1371/journal.pone.0241570 (PMC7657484; doi:10.1371/journal.pone.0241570)
Supplement: S1 Table — (DOCX) [file pone.0241570.s002.docx]

| Data: Paper | Access: DOI |
| --- | --- |
| Ålmgren M, Lennerling A, Lundmark M, Forsberg A. The meaning of being in uncertainty after heart transplantation – an unrevealed source to distress. Eur J Cardiovasc Nurs. 2017;16(2):167-74. | https://doi.org/10.1177/1474515116648240 |
| Ålmgren M, Lennerling A, Lundmark M, Forsberg A. Self-efficacy in the context of heart transplantation – a new perspective. J Clin Nurs. 2017;26(19-20):3007-17. | https://doi.org/10.1111/jocn.13647 |
| .  Dabbs ADV, Hoffman LA, Swigart V, Happ MB, Dauber JH, McCurry KR, et al. Striving for normalcy: symptoms and the threat of rejection after lung transplantation. SocSci Med. 2004;59(7):1473-84. | https://doi.org/10.1016/j.socscimed.2004.01.013 |
| Evangelista LS, Doering L, Dracup K. Meaning and life purpose: the perspectives of post-transplant women. Heart Lung. 2003;32(4):250-7 | https://doi.org/10.1016/s0147-9563(03)00042-6 |
| Flynn K, Daiches A, Malpus Z, Yonan N, Sanchez M. ‘A post-transplant person’: Narratives of heart or lung transplantation and intensive care unit delirium. Health.2014;18(4):352-68. | https://doi.org/10.1177/1363459313501356 |
| Ivarsson B, Ekmehag B, Sjöberg T. Patients’ experiences of information and support during the first six months after heart or lung transplantation. Eur J Cardiovasc Nurs. 2013;22(11-12):400-6. | https://doi.org/10.1177/1474515112466155 |
| Ivarsson B, Ekmehag B, Sjöberg T. Heart or lung transplanted patients' retrospective views on information and support while waiting for transplantation. J Clin Nurs. 2013;22(11- 12):1620-8. | https://doi.org/10.1111/j.1365-2702.2012.04284.x |
| Kaba E, Thompson DR, Burnard P. Coping after heart transplantation: a descriptive study of heart transplant recipients’ methods of coping. J Adv Nurs. 2000;32(4):930-6. 51. | https://doi.org/10.1046/j.1365-2648.2000.t01-1-01  558.x |
| Kaba E, Thompson DR, Burnard P, Edwards D, Theodosopoulou E. Somebody else's heart inside me: a descriptive study of psychological problems after a heart transplantation. Issues Ment Health Nurs. 2005;26(6):611-25. | https://doi.org/10.1080/01612840590959452 |
| Lawrence K, Stilley CS, Olshansky E, Bender A, Webber SA. Further exploration: Maturity and adherence in adolescent and young adult heart transplant recipients. Prog Transplant. 2008;18(1):50-4. | https://doi.org/10.1177/152692480801800110 |
| Lundmark M, Lennerling A, Almgren M, Forsberg A. Recovery after lung transplantation from a patient perspective – proposing a new framework. J Adv Nurs. 2016;72(12):3113-24 | https://doi.org/10.1111/jan.13058 |
| Macdonald K. Living in limbo - patients with cystic fibrosis waiting for transplant. BrJ Nurs. 2006;15(10):566-72. | https://doi.org/10.12968/bjon.2006.15.10.21134 |
| Mauthner OE, De Luca E, Poole JM, Abbey SE, Shildrick M, Gewarges M, et al.  Heart transplants: Identity disruption, bodily integrity and interconnectedness. Health. 2015;19(6):578-94. | https://doi.org/10.1177/1363459314560067 |
| Moloney S, Cicutto L, Hutcheon M, Singer L. Deciding about lung transplantation: Informational needs of patients and support persons. Prog Transplant. 2007;17(3):183-92. | https://doi.org/10.1177/152692480701700305 |
| Neukom M, Corti V, Boothe B, Boehler 636 A, Goetzmann L. Fantasized recipient–donor relationships following lung transplantations: A qualitative case analysis based on patient narratives. Int J Psychoanal. 2012;93(1):117-37.2015;19(6):578-94. 2015;19(6):578-94. | https://doi.org/10.1111/j.1745-8315.2011.00496.x |
| Nilsson M, Persson L-O, Forsberg A. Perceptions of experiences of graft rejection among organ transplant recipients striving to control the uncontrollable. J Clin Nurs. 2008;17(18):2408-17. | https://doi.org/10.1111/j.1365-2702.2008.02364.x |
| O’Brien GM, Donaghue N, Walker I, Wood CA. Deservingness and gratitude in the context of heart transplantation. Qual Health Res. 2014;24(12):1635-47. | https://doi.org/10.1177/1049732314549018 |
| Palmar-Santos AM, Pedraz-Marcos A, Zarco-Colón J, Ramasco-Gutiérrez M, García- Perea E, Pulido-Fuentes M. The life and death construct in heart transplant patients. Eur J Cardiovasc Nurs. 2019;18(1):48-56. 39 | https://doi.org/10.1177/1474515118785088 |
| Peyrovi H, Raiesdana N, Mehrdad N. Living with a heart transplant: A phenomenological study. Prog Transplant. 2014;24(3):234-41. | https://doi.org/10.7182/pit2014966 |
| Poole J, Ward J, DeLuca E, Shildrick M, Abbey S, Mauthner O, et al. Grief and loss for patients before and after heart transplant. Heart Lung. 2016;45(3):193-8. | https://doi.org/10.1016/j.hrtlng.2016.01.006 |
| Sadala MLA, Stolf NAG. Heart transplantation experiences: a phenomenological approach. J Clin Nurs. 2008;17(7b):217-25. | https://doi.org/10.1111/j.1365-2702.2007.02206.x |
| Sanner MA. Transplant recipients’ conception of three key phenomena in transplantation: the organ donation, the organ donor, and the organ transplant. Clin Transplant. 2003;17:391-400. | https://doi.org/10.1034/j.1399-0012.2003.00065.x |
| Thomsen D, Jensen BØ. Patients’ experiences of everyday life after lung transplantation. J Clin Nurs. 2009;18(24):3472-9. | https://doi.org/10.1111/j.1365-2702.2009.02828 |
| Waldron R, Malpus Z, Shearing V, Sanchez M, Murray CD. Illness, normality and identity: the experience of heart transplant as a young adult. Disabil Rehabil.2017;39(19):1976-82. | https://doi.org/10.1080/09638288.2016.1213896 |
